# Privacy, Trust, and Data Sharing in Web-Based and Mobile Research: Participant Perspectives in a Large Nationwide Sample of Men Who Have Sex With Men in the United States

**DOI:** 10.2196/jmir.9019

**Published:** 2018-07-04

**Authors:** H Jonathon Rendina, Brian Mustanski

**Affiliations:** ^1^ Department of Psychology Hunter College The City University of New York New York, NY United States; ^2^ Doctoral Program in Health Psychology and Clinical Science The Graduate Center The City University of New York New York, NY United States; ^3^ Department of Medical Social Sciences Feinberg School of Medicine Northwestern University Chicago, IL United States

**Keywords:** data privacy, data sharing, research trust, mobile research, research ethics, men who have sex with men, gay and bisexual men

## Abstract

**Background:**

Modern research is heavily reliant on online and mobile technologies, which is particularly true among historically hard-to-reach populations such as gay, bisexual, and other men who have sex with men (GBMSM). Despite this, very little empirical research has been published on participant perspectives about issues such as privacy, trust, and data sharing.

**Objective:**

The objective of our study was to analyze data from an online sample of 11,032 GBMSM in the United States to examine their trust in and perspectives on privacy and data sharing within online and mobile research.

**Methods:**

Participants were recruited via a social networking site or sexual networking app to complete an anonymous online survey. We conducted a series of repeated measures analyses adjusted for between-person factors to examine within-person differences in the following: (1) trust for guarding personal information across different venues (eg, online research conducted by a university vs. an online search engine); (2) privacy concerns about 12 different types of data for three distinct data activities (ie, collection by app owners, anonymous selling to third parties, and anonymous sharing with researchers); and (3) willingness to share those 12 different types of data with researchers. Due to the large sample size, we primarily reported measures of effect size as evidence of clinical significance.

**Results:**

Online research was rated as most trusted and was more trusted than online and mobile technology companies, such as app owners and search engines, by magnitudes of effect that were moderate-to-large (η_partial_^2^=0.06-0.11). Responding about 12 different types of data, participants expressed more concerns about data being anonymously sold to third-party partners (mean 7.6, median 10.0) and fewer concerns about data being collected by the app owners (mean 5.8, median 5.0) or shared anonymously with researchers (mean 4.6, median 3.0); differences were small-to-moderate in size (η_partial_^2^=0.01-0.03). Furthermore, participants were most willing to share their public profile information (eg, age) with researchers but least willing to share device usage information (eg, other apps installed); the comparisons were small-to-moderate in size (η_partial_^2^=0.03).

**Conclusions:**

Participants reported high levels of trust in online and mobile research, which is noteworthy given recent high-profile cases of corporate and government data security breaches and privacy violations. Researchers and ethical boards should keep up with technological shifts to maintain the ability to guard privacy and confidentiality and maintain trust. There was substantial variability in privacy concerns about and willingness to share different types of data, suggesting the need to gain consent for data sharing on a specific rather than broad basis. Finally, we saw evidence of a privacy paradox, whereby participants expressed privacy concerns about the very types of data-related activities they have likely already permitted through the terms of the apps and sites they use regularly.

## Introduction

Since the development of the “World Wide Web” nearly three decades ago, the diversity and usage of available online and mobile technologies have proliferated rapidly, resulting in a shift in the landscape of their use among various populations. Among gay, bisexual, and other men who have sex with men (GBMSM), these shifts have been evident in the use of these technologies for sexual networking, which has developed from online computer chat rooms to mobile geosocial networking applications (ie, “apps”) to identify potential partners by various characteristics and categorize them by distance [1]. Evidence suggests that a large number of GBMSM use these technologies to locate sexual partners [2]. As a result of their popularity, researchers have leveraged these technologies to reach and recruit GBMSM—who have historically been a hidden or hard-to-reach population [3]—into formative and intervention studies, particularly on a range of HIV prevention and treatment topics [4-14]. Although most research, both with and without such technologies, has traditionally focused on HIV, the focus is gradually broadening [15], given that GBMSM are part of the broader population of sexual and gender minorities and are now recognized by the US National Institutes of Health as a “health disparity population” [16]. As the GBMSM-focused research agenda broadens, it is also likely to shift more toward online and mobile methodologies because of their popularity. However, this surge in research using technology brings with it novel methodological and ethical considerations.

When making decisions regarding the ethical implications of online and mobile research, researchers and review boards are charged with evaluating and minimizing risk to participants, but rapidly evolving technological advances have made it difficult to keep pace [17,18]. Researchers and ethical review boards experience several issues related to human subjects’ protections in online and mobile research that are either unique or different from those encountered in traditional research, including issues of informed consent, privacy/confidentiality, data security, and ownership of and access to data [19-25]. In recent years, there has been a substantial increase in the number of scientific papers reviewing the state of science, empirically evaluating or discussing the implications of privacy, security, and confidentiality in online and mobile research [17,19,20,22-29]. Besides risks that offline and online research share, one of the primary forms of risk posed by online and mobile research is that of informational risk [20,30], which is risk that research might lead to unintended creation, tracking, or sharing of data with third parties or interception of data by other audiences [23]. For example, many individuals—whether potential participants, researchers themselves, or ethics reviewers—are unaware of the extent to which third-party marketing firms could track and store information about individuals’ internet behaviors (eg, clicking on an ad for a research study) to create a complex profile of individuals for advertising purposes [20]. To the extent that such data, even if minimally detailed, are collected by app owners without the knowledge of researchers or participants, issues can arise about understanding and protecting the privacy and confidentiality of participants, thus potentiating research mistrust.

In addition to understanding the technical and legal aspects of risks when using online and mobile technologies, it is important to understand and weigh participants’ perspectives on trust, privacy, and data sharing. Regarding issues of privacy and confidentiality when using online and mobile technology for personal rather than research purposes, views continue to develop among the general public together with the changing technological landscape [31-35]. For example, data on 461 adults in the United States collected by Pew Research Center suggested that people weigh tradeoffs between disclosing personal information and the benefits of doing so; more than half (56.8%, 262/461) considered it acceptable to use a health information website that their doctor would upload their health data to as long as it was secure (ie, high benefits and low likelihood of disclosures), whereas only one-third (33.2%, 153/461) considered it acceptable to use a social media site that would use their profile data to deliver targeted advertising [36]. Subsequent Pew data highlighted the general public’s trust in online and mobile companies that they regularly use; data from 1040 US adults in 2016 suggested that 65.3% (605/926) were somewhat or very confident that their email providers adequately safeguard the privacy of their data, although this figure was only 47.2% (314/665) for social media sites that they used [37].

Compared with the available data on participants’ perspectives on privacy within the technologies they use for personal reasons, fewer published studies are available regarding participants’ perspectives on these issues in online and mobile research. Nonetheless, the available data suggest equally nuanced and developing views. One study showed that people preferred online methods over traditional means of research and considered online research to be *more* private than traditional in-person methods, although submitting sensitive health and personal information emerged as a concern [38]. Another recent study reported that concerns about privacy and confidentiality in online and mobile research are diverse and often contextually specific, varying across individuals, as well as by the type of data, the context of data collection, and the purpose of data collection or usage [39,40]. Echoing the findings outside the research context, these studies suggested that participants value control over whether and how data are used.

Although the data above highlight participants’ viewpoints regarding privacy in online and mobile research from the general public and despite growing literature on methodological issues related to online and mobile research with GBMSM [1,4,6,8,9], we are unaware of any published research that has examined the perceptions of privacy in online and mobile research specific to GBMSM. Given the growing use of online and mobile technologies within research with GBMSM and the relatively unique technologies available to and used by GBMSM, it is imperative to understand their views about the risks and benefits of technology-based research. We believe such data will be of use to future researchers as they design technology-based studies, consider industry partnerships to conduct research, and weigh the risks and benefits of such designs.

This study was designed to fill the noted gaps in the literature on GBMSM perspectives on trust, privacy, and data sharing in online and mobile research and to achieve three aims. First, we sought to understand trust in online and mobile research compared with that in the use of online and mobile technologies for everyday purposes. Thus, we compared levels of trust for guarding personal information—defined broadly—across numerous sources that collect such data (eg, an online research study vs. a social networking website). Second, we sought to better understand which specific types of data caused participants more and less concern about privacy. We compared the extent of privacy concerns endorsed for three distinct practices within a hypothetical app—collection and storage of the data by app developers, sale of data anonymously to third-party partners, and sharing of data anonymously with researchers—across a range of unique types of personal data. Third, we sought to examine willingness to have different types of app-generated data shared with researchers. Using the same unique types of personal data from the second aim, we compared hypothetical willingness to provide consent to have an app developer/owner share these different types of data anonymously with researchers.

## Methods

In this study, data were reported from an extensive nationwide survey of GBMSM conducted over a 4-week period between May and June 2017.

### Participants and Procedures

Between May 17, 2017 and June 10, 2017, we used advertisements to enroll GBMSM from two venues—one of the most popular geotargeted sexual networking apps for GBMSM and one of the most popular social networking websites for the general population. The sexual networking app pushed the advertisement as a message to the chat inboxes of all users in the United States on Friday, May 19, 2017, which remained for 7 days, unless deleted sooner. On the social networking site, we used targeted banner advertisements for approximately 4 weeks that could show up in one of the two ways—a static ad on the right-hand pane of the website or an ad that resembled a normal post as users scrolled through their feeds. We targeted the social networking site ads to people who were men, residing in the United States, aged ≥18 years, and believed to be GBMSM based on either a same-sex interest listed on their profile or a range of relevant “likes” (eg, gay pride, lesbian, gay, bisexual, and transgender, LGBT, community, gay bar, and same-sex marriage). Both ads comprised a background image (the social networking site: 2 clothed men on a bed kissing; the sexual networking app, 2 bare torsos embracing) and brief text, including that they could “enter to win a $50 Amazon.com gift card” and that there was “no participation necessary” to enter the random drawing.

Upon clicking on the ad, the participants were informed that the survey would take approximately 10-15 min to complete and provided the option to begin immediately or enter their email address to receive a link to complete later. Upon beginning the survey—whether immediately or through the emailed link—participants were provided with a brief online consent form and given the options of providing consent, declining, or declining with the option to receive instructions for entering the random gift card drawing. During consent, participants were informed about a 1 in 100 chance of receiving a $50 gift card. Those who subsequently declined consent were provided instructions should they want to enter the drawing; conversely, participants who completed the survey and were interested in entering the drawing were redirected to a separate survey in which they were required to enter an email address that was not linked in any way to their data. During the first few questions of the survey, participants were screened for eligibility, which was defined as follows: (1) age ≥18 years; (2) residing in the United States; (3) having had same-sex sexual activity within the past year; and (4) identifying as male (including both cisgender and transgender males). Those who were ineligible were informed after the first few questions, and the survey subsequently ended. The study protocol was reviewed and approved by the Human Research Protections Program of The City University of New York (New York, NY, USA).

We followed a protocol based on standards within the literature [41] for removing potentially duplicate cases while erring on the side of keeping rather than removing data in cases where a determination could not be made. In particular, we first identified potential duplicates based on birth month and year, zip code, HIV status, and race/ethnicity; all cases sharing those features in common were manually examined, focusing on responses to other questions such as education, employment, and partner status, as well as device and browser information and the survey duration.

### Measures

We collected all measures for this study as self-reported items and scales within the one-time online survey. The item content was developed in part by consulting the terms of service and privacy policy for two social networking (ie, Facebook and Facebook Messenger) and two sexual networking (ie, Grindr and Scruff) apps in late 2016. In addition, we examined the types of personal information and data discussed within those agreements and the usage provided for within the agreements to develop three primary data activities described in the measure below (ie, data collection, anonymous sale of data, and anonymous sharing of data). Likewise, we used the sites and apps to create a list of the types of personal information (ie, data) that are likely to be gathered and/or generated by developers. After obtaining the complete draft of the measures, we invited a group of 20 adult GBMSM in the New York City area to participate in an in-person community feedback session; all participants were provided with a copy of the measures, and we reviewed both the study procedures (eg, recruitment and compensation) and the item content with them to gather their feedback. We received and followed numerous suggestions to improve clarity, reduce length, and minimize burden. For example, from a list of at least 15 different types of data, community members noted that they were not all meaningfully distinct; thus, the list was condensed to form broader categories in some cases. Similarly, we implemented suggestions for improved wording. The final version of the measures was based on this feedback and a review by field experts from Fordham University’s HIV Prevention and Substance Use Research Ethics Training Institute (New York, NY), as described later (see the Online Supplementary Material for more details).

#### Demographic Characteristics

Participants responded to items inquiring about various demographic characteristics, including age, zip code (which was converted to geographic region), relationship status, sexual orientation, and race/ethnicity.

#### Trust to Safeguard Personal Information

All participants received the following instructions:

“We are interested in knowing more about how much you trust various organizations and businesses to protect the privacy and confidentiality of the data they collect on you. Please assume you are being asked to provide similar information to each. How much do you trust that each of the following sources would guard the privacy and confidentiality of your personal information?”

Following this, they were presented with a list of nine different types of online and mobile venues in which personal information could be collected and asked to rate their trust on a scale from 1 (*Not at all trusting*) to 4 (*Very trusting*).

#### Concerns About Privacy Threats

We presented the participants with a vignette describing a hypothetical new app with various features. Then, a series of 12 types of personal information were presented and participants were asked, for each, whether the following activities concerned them as a threat to their privacy: (1) app owners privately collecting and storing these data; (2) app owners selling these data anonymously to third-party marketing groups; and (3) app owners sharing these data anonymously with researchers. Participants were asked to check which, if any, of the three activities concerned them separately for each of the 12 types of personal information (ie, a total of 36 dichotomous responses).

#### Data Sharing With Researchers

Finally, we presented the participants with the same 12 types of personal information from the prior measure and the following instructions:

“Within this study, we are not gathering any data on you from any apps or sites that you use. However, please imagine we were interested in connecting data collected by the app with the data you provided in this survey. Which of the following would you give us permission to gather anonymously from the app owners to link with your survey data?”

Participants rated their willingness to provide permission for each on a scale from 1 (*Definitely not*) to 4 (*Definitely*).

### Data Analysis

All analyses were performed in SPSS 24 (IBM Corporation; Amonk, New York, United States). To inform future online recruitment efforts, we began our analyses by characterizing the sociodemographic characteristics of the sample and comparing them across the two recruitment venues using chi-square tests of independence. To address the first aim regarding the comparisons of trust for guarding personal information across nine different sources, we iteratively conducted a series of 36 repeated measures analysis of variance (RMANOVA) models examining each pair of ratings while adjusting for relevant between-person characteristics (ie, recruitment source, race, HIV status, and age); we specified an interaction for each between-person factor with the within-person factor but not among the between-person factors. We reported the η_partial_^2^ effect sizes for the within-person main effect as evidence of the magnitude of each comparison. To address the second aim regarding privacy concerns raised about 12 different types of app-related data across 3 different data activities (ie, the app collecting the data, the app anonymously selling the data, and the app anonymously sharing the data with researchers), we assessed the prevalence of indicating each was a concern by examining the frequency and proportion of “yes” responses across the 36 dichotomous indicators. We also calculated a sum score for the total number of types of data that raised concerns for participants for each of the 3 data activities and compared the 3 sum scores to one another in an RMANOVA that was consistent with the prior set of analyses with two exceptions—all 3 scores were compared simultaneously rather than in pairs, and we used a simple contrast to test differences between the three, using sharing with researchers as the referent group. Finally, to address the third aim regarding which types of app-related data participants would hypothetically be willing to provide explicit permission to have shared with researchers, we used the same 12 types of data asked about in the second aim and used a series of 66 pairwise RMANOVAs consistent with the first set of analyses to compare within-person differences among the 12 ratings adjusted for the relevant between-person factors.

Across all analyses, the primary goal was to examine patterns in the data descriptively using effect sizes rather than search for statistical significance, particularly because of the large sample size. Furthermore, we reported the η_partial_^2^ effect size as small (0.01), medium/moderate (0.06), and large (0.14) in size [42]. Nonetheless, for statistical comparisons, we reported statistical significance for those findings that reached a threshold of *P*<.001 to reduce the likelihood of type II error because of multiple comparisons.

We conducted an experimental manipulation to test whether providing a rationale for each of the 3 activities within the “*Concerns about privacy threats* ” measure would influence trust. Specifically, participants were randomized to receive either a description of the 3 activities with no rationale or the same description with rationale added (eg, for the app owners collecting the information, rationale added was “to improve, tailor, and develop the services you use”). As results suggested nonsignificant and extremely small (Cohen *d*<.05) differences between groups, all results are presented irrespective of the experimental condition.

## Results

Upon reaching the landing page of the survey from the advertisement, 80.4% (21,942/27,291) of participants agreed to be immediately linked to the survey, 17.1% (4677/27,291) opted to receive an email and complete the survey at a later time, and 2.5% (672/27,291) opted not to take the survey. Subsequently, 18,909 reached the consent form, of whom 94.9% (17,954/18,909) provided consent, 1.4% (262/18,909) declined consent, and 3.7% (693/18,909) requested instructions on how to enter the drawing without completing the survey. Of 17,954 who provided consent, 7.4% (1335/17,954) did not provide sufficient data to determine eligibility, 11.5% (2068/17,954) were deemed ineligible, 19.4% (3487/17,954) were eligible but only partially completed the survey, and 61.6% (11,064/17,954) completed the survey in its entirety. Among those who reached the consent form, the completion rates were similar for those who began the survey from the social networking site (56.6%, 2193/3874) and the sexual networking app (59.0%, 8871/15,035). Finally, of the completed surveys, we eliminated 30 completed surveys that were duplicate responses of previously completed surveys, resulting in a final analytic sample of 11,032 GBMSM in the United States.

Table 1 summarizes the sociodemographic characteristics of the analytic sample with comparisons by recruitment source—nearly one-fifth (19.6%, 2166/11,032) were recruited from the social networking site and the remainder (80.4%, 8866/11,032) were enrolled from the sexual networking app. The sample was diverse regarding race/ethnicity, with nearly half (46.2%, 5102/11,032) being men of color. Most of the sample identified as cisgender male (98.5%, 10,869/11,032), and gay or queer (81.9%, 9045/11,032) and the majority reported being HIV-negative (75.0%, 8275/11,032); we observed diversity in employment, educational experiences, and geographic regions. In addition, we observed significant differences between the 2 recruitment sources regarding race/ethnicity, gender identity, sexual identity, employment status, and geographic region; the sexual networking app comprised more men of color, fewer transgender males, more nongay identified men, more men who were working full-time, and fewer men from the South. The sample ranged in the 18-80 years of age, with an average age of 32.6 (SD 12.0; median 29.0) years, with the social networking site (mean 33.3, SD 14.3) being 1 year older, on average, than the sexual networking app (mean 32.4, SD 11.3).

Table 2 presents the ratings of trust to guard personal information by source, with corresponding within-person comparisons across all sources reported as η_partial_^2^ effect sizes. As evident within the unadjusted means, participants rated the 3 types of online research studies with a high degree of trust for guarding personal information—the median rating for each was a 3 on a range of 1-4 with minimal differences between them. The next most trusted source was the partnership between researchers and a mobile app for GBMSM, which exhibited minimal differences in trust ratings from those of the 3 types of online research and medium-to-large differences from each of the 5 types of online and mobile companies. The mobile app designed for GBMSM was rated much lower than the four types of online research and slightly higher than the online and mobile technologies for the general public based on the unadjusted means; however, the adjusted within-person comparisons revealed inconsequentially small differences in rating between the GBMSM-specific app and each of the 3 types of online and mobile companies for the general public, which also had minimal differences from one another.

Table 3 presents the prevalence of data concerns by each type of data and data activity (ie, data collection, anonymous data sale to third parties, and anonymous data sharing with researchers). Here, two trends are worth noting. First, across the 3 activities, there was diversity in terms of which *types* of data participants were concerned about—the most widely endorsed types of data that concerned participants were device data, such as global positioning system (GPS) information and information about other apps installed on the phone, whereas the least endorsed were about usage of the app such as how often one logged in or whether they participated in any app-based health promotion campaigns. Second, regardless of the *type* of data, there was a trend about the data *activities* that were the most concerning, with a marked number of participants endorsing a concern about the anonymous sale of their data to third-party partners and the lowest numbers endorsing concern about the anonymous sharing of their data with researchers. Notably, across each type of data, more participants expressed concern about the app collecting their data in the first place than did about the anonymous sharing of their data with researchers. Table 3 also presents the average number of types of data endorsed as concern for each of the 3 activities. In within-person comparisons, we found that all three were significantly different from one another (η_partial_^2^=0.01; *P*<.001), with a small-to-moderate difference between anonymous data sharing with researchers and anonymous data selling to third parties (η_partial_^2^=0.03) and a small difference between sharing with researchers and collection of the data themselves (η_partial_^2^=0.01).

**Table 1:** Sociodemographic characteristics and comparisons by the recruitment source.

Characteristics		Full sample (N=11,032), n (%)	Social networking site (n=2166), n (%)	Sexual networking app (n=8866), n (%)	χ^2^ (*df*)
**Race/Ethnicity**					216.4 (4)^a^
	Black	1100 (10.0)	107 (4.9)	993 (11.2)	
	Latino	2409 (21.8)	344 (15.9)	2065 (23.3)	
	White	5930 (53.8)	1456 (67.2)	4474 (50.5)	
	Multiracial	808 (7.3)	142 (6.6)	666 (7.5)	
	Other	785 (7.1)	117 (5.4)	668 (7.5)	
**Gender Identity**					198.9 (1)^a^
	Cisgender male	10869 (98.5)	2063 (95.2)	8806 (99.3)	
	Transgender male	163 (1.5)	103 (4.80)	60 (0.7)	
**Sexual Identity**					33.8 (3)^a^
	Gay, queer, or homosexual	9045 (82.0)	1862 (86.0)	7183 (81.0)	
	Bisexual	1802 (16.3)	275 (12.7)	1527 (17.2)	
	Heterosexual	46 (0.4)	2 (0.1)	44 (0.5)	
	Other	139 (1.3)	27 (1.2)	112 (1.3)	
**Employment Status**					57.6 (3)^a^
	Full-time	5990 (54.3)	1038 (47.9)	4952 (55.9)	
	Part-time	2505 (22.7)	528 (24.4)	1977 (22.3)	
	On disability	655 (5.9)	180 (8.3)	475 (5.4)	
	Unemployed	1882 (17.1)	420 (19.4)	1462 (16.5	
**Educational Attainment**					0.6 (3)
	High school, GED^b^, or less	2395 (21.7)	469 (21.7)	1926 (21.7)	
	Some college	4908 (44.5)	975 (45.0)	3933 (44.4)	
	4-year college degree	2434 (22.1)	465 (21.5)	1969 (22.2)	
	Postgraduate degree	1295 (11.7)	257 (11.9)	1038 (11.7)	
**HIV Status**					9.0 (2)
	Negative	8275 (75.0)	1679 (77.5)	6596 (74.4)	
	Positive	1837 (16.7)	326 (15.1)	1511 (17.0)	
	Unknown	920 (8.3)	161 (7.4)	759 (8.6)	
**Geographic Region**					20.3 (4)^a^
	Northeast	2089 (18.9)	400 (18.5)	1689 (19.1)	
	South	2045 (18.5)	469 (21.7)	1576 (17.8)	
	Midwest	3777 (34.2)	699 (32.3)	3078 (34.7)	
	West	3034 (27.5)	587 (27.1)	2447 (27.6)	
	Other/Unknown	87 (0.8)	11 (0.5	76 (0.9)	

^a^*P*<.001

^b^GED: General Equivalency Diploma.

**Table 2:** Within-person comparisons of trust to guard the privacy of personal information reported as η_partial_^2^ effect sizes. Results are reported as η_partial_^2^ effect sizes for the difference between the two means adjusted for demographic covariates (eg, unadjusted means, medians, and standard deviations are presented in the far right columns to ease interpretation of the comparisons). Response options ranged from 1 (*not at all trusting*) to 4 (*very trusting*).

Source	1	2	3	4	5	6	7	8	9	Mean (SD)	Median
1. Online research study by researchers at a university	—									2.82 (0.84)	3.00
2. Online research study by an LGBT^a^ community center	0.00	—								2.87 (0.82)	3.00
3. Online research study by government health agency	0.00^b^	0.00^b^	—							2.81 (0.96)	3.00
4. Mobile networking app for GBMSM^c^	0.08^b^	0.10^b^	0.09^b^	—						2.03 (0.84)	2.00
5. Mobile networking app for the general public	0.10^b^	0.11^b^	0.11^b^	0.00^b^	—					1.81 (0.81)	2.00
6. Online shopping website	0.06^b^	0.07^b^	0.08^b^	0.00	0.00	—				1.84 (0.91)	2.00
7. Online email website	0.06^b^	0.06^b^	0.07^b^	0.00	0.00^b^	0.00	—			1.83 (0.90)	2.00
8. Online search engine	0.06^b^	0.07^b^	0.08^b^	0.00	0.00^b^	0.00	0.00	—		1.83 (0.90)	2.00
9. Research study by researchers at a university in collaboration with mobile networking app for GBMSM	0.00	0.00	0.01^b^	0.09^b^	0.10^b^	0.06^b^	0.06^b^	0.06^b^	—	2.69 (0.87)	3.00

^a^LGBT: lesbian, gay, bisexual, and transgender.

^b^*P*<.001.

^c^GBMSM: gay, bisexual, and other men who have sex with men.

**Table 3:** Prevalence of privacy concerns by type of data and data activity. Numbers and percentages correspond to those participants who endorsed each item as a concern.

Type of data	App owners collecting, n (%)		App owners anonymously selling to third parties, n (%)		App owners anonymously sharing with researchers, n (%)	
Public profile information (eg, age and height)	5523 (50.1)		7302 (66.2)		4081 (37.0)	
Account information (eg, birthdate and zip code)	5418 (49.1)		7500 (68.0)		4106 (37.2)	
Match information (eg, HIV status and dating interests)	5039 (45.7)		7016 (63.9)		4090 (37.1)	
Mobile device information (eg, operating system)	5187 (47.0)		6901 (62.6)		4258 (38.6)	
Interaction information (eg, demographics of chat partners)	5251 (47.6)		6964 (63.1)		4143 (37.6)	
App usage information (eg, login frequency)	4843 (43.9)		6459 (58.5)		3783 (34.3)	
Health campaign participation information (eg, HIV test reminders)	4713 (42.7)		6540 (59.3)		3867 (35.1)	
Device GPS^a^ information (eg, login locations)	6020 (54.6)		7469 (67.7)		4735 (42.9)	
Device usage information (eg, other apps installed)	6337 (57.4)		7563 (68.6)		5138 (46.6)	
App advertising information (eg, ad clicks)	5047 (45.7)		6890 (62.5)		4123 (37.4	
Third-party advertiser information (eg, service utilization)	5165 (46.8)		6880 (62.4)		4143 (37.6	
App-generated information (eg, advertising profiles)	5016 (45.5)		6810 (61.7)		4043 (36.6)	
Total number of concerns (range: 0-12), mean (median)	5.8 (5.0)		7.6 (10.0)		4.6 (3.0)	

**Table 4:** Willingness to share various data types with researchers and within-person comparisons between each reported as η_partial_^2^ effect sizes. Results are reported as η_partial_^2^ effect sizes for the difference between the two means adjusted for demographic covariates (eg, unadjusted means, medians, and standard deviations are presented in the far right columns to ease interpretation of the comparisons). Responses ranged from 1 (*definitely not*) to 4 (*definitely*).

Type of data	1	2	3	4	5	6	7	8	9	10	11	12	Mean (SD)	Median
1. Public profile information	—												2.85 (0.93)	3.00
2. Account information	0.05^a^	—											2.35 (1.00)	2.00
3. Match information	0.01^a^	0.02^a^	—										2.65 (0.97)	3.00
4. Mobile device information	0.03^a^	0.00	0.01^a^	—									2.27 (1.03)	2.00
5. Interaction information	0.02^a^	0.01^a^	0.01^a^	0.00^a^	—								2.35 (0.99)	2.00
6. App usage information	0.01^a^	0.02^a^	0.00	0.01^a^	0.01^a^	—							2.48 (0.98)	3.00
7. Health campaign participation	0.00^a^	0.02^a^	0.00	0.02^a^	0.01^a^	0.00	—						2.58 (0.98)	3.00
8. Device GPS^b^ information	0.06^a^	0.01^a^	0.04^a^	0.01^a^	0.02^a^	0.04^a^	0.05^a^	—					2.08 (1.02)	2.00
9. Device usage information	0.06^a^	0.01^a^	0.04^a^	0.01^a^	0.02^a^	0.04^a^	0.05^a^	0.00	—				1.92 (1.00)	2.00
10. App advertising information	0.03^a^	0.00^a^	0.01^a^	0.00	0.00	0.01^a^	0.01^a^	0.02^a^	0.02^a^	—			2.24 (0.99)	2.00
11. Third-party advertiser information	0.03^a^	0.00^a^	0.01^a^	0.00	0.00^a^	0.01^a^	0.02^a^	0.01^a^	0.02^a^	0.00	—		2.21 (0.99)	2.00
12. App-generated information	0.02^a^	0.01^a^	0.00^a^	0.00^a^	0.00	0.00^a^	0.01^a^	0.03^a^	0.03^a^	0.00^a^	0.01^a^	—	2.33 (0.99)	2.00

^c^*P*<.001.

^a^GPS: global positioning system.

Besides knowing which types of data collection, sale, and sharing are of concern as a threat to participant’s privacy, we were also interested in determining which types of data they would give explicit permission to researchers to request from app owners. Table 4 presents the average willingness expressed by participants for each type of data, which were similar to those examined in the prior set of analyses. We observed a range of willingness across the 12 types of data with adjusted within-person differences between different types of data ranging from very small (η_partial_^2^<0.01) to medium (η_partial_^2^= 0.06); the majority of participants were willing to share 4 types of data and unwilling to share the other 8 (ie, 4 had median values of 3.0 corresponding to probable willingness). Consistent with the previous aim’s findings on which types of data represented a privacy concern, participants were least willing to share those data that were generated by their devices such as GPS and other apps installed, whereas they were most willing to share general app information such as public profile and match survey data, as well as app usage statistics and health campaign participation. In fact, the largest differences were in comparing the public profile information with device GPS data (η_partial_^2^= 0.06) and device usage information (η_partial_^2^= 0.06).

## Discussion

### Primary Findings

We analyzed data from an online sample of 11,032 GBMSM across the United States to examine participant perspectives on the issues of trust, privacy, and data sharing in online and mobile research. In analyses that were adjusted for relevant between-person differences (including the recruitment site), we found that trust in online research was greater than trust in online and mobile platforms for personal use, such as social and sexual networking apps or various types of websites. When focusing on 12 different types of data that could be gathered by a hypothetical sexual networking app, participants expressed the least concerns about privacy when such data were going to be shared anonymously with researchers and the most concern when these data were going to be sold anonymously to third parties; the actual collection of the data by the app owners raised an intermediate level of concern. Finally, reviewing the same 12 types of data, we examined which types of data participants would be willing to share within future research studies—participants were most willing to share information they disclose publicly within the app (such as profile information on characteristics like age and height) and least willing to share information that could be collected by the app automatically (such as GPS location or device usage information).

We found overall moderate levels of trust within online research studies, with little difference based on the type of organization conducting the research. In this study, approximately two-thirds of GBMSM trusted or highly trusted online and mobile research compared with one-quarter who trusted GBMSM-specific networking apps and approximately 18% who trusted networking apps used by the general public. Although not asked in exactly the same way, these findings suggested lower levels of trust in this sample than those in a previous Pew poll [37], which could be due to the population or due to more general shifts that occurred in the year that passed between the 2 studies. Although we asked about trust in online research across three different types of organizations (ie, LGBT community center, a university, and a government agency), participants in this study did not appear to differentiate between online research done by these three different groups and reported similar levels of trust for each. Particularly for researchers to understand, participants expressed greater trust in research than in many of the online and mobile technology companies and services they use on a regular—if not daily—basis. With the proliferation of public-private partnerships and collaborations between research organizations and these service providers, it is critical to consider how this might affect trust within both sources. Efforts to maintain trust by promoting transparency in research practices within such partnerships might prove critical. For example, getting informed consent before having data shared anonymously could be the best practice, even when such permission has already been granted within the terms of service for the app or site and research activities might qualify for a waiver of informed consent based on the federal criteria for human subjects review exemptions if data are transmitted anonymously.

In this study, participants expressed concern about several data collection, selling, and sharing activities. These findings are consistent with a study on the *privacy paradox* [43], which suggests that individuals’ concerns about privacy are discrepant with their own privacy practices (eg, privacy settings). In this case, the paradox results from participants expressing concerns about the very types of data collection, selling, and sharing that they have likely already agreed to within the terms of service and privacy policies of the very apps and sites they regularly use and from which they were enrolled. Also, somewhat unexpectedly, more participants expressed concern about the actual collection of these various types of data by the app owners than they did about the anonymous sharing of the same data with researchers (an act that would be impossible without the apps first collecting these data). One potential explanation for this set of findings could be that the data remain connected to participants’ identities for the app owners, whereas they were specifically referenced as anonymous when sharing with researchers. Another possibility is that this higher willingness to share data with researchers than have it collected in the first place by the app owners is due to the higher levels of trust in research that were observed within the analyses for the first aim of this study. Nevertheless, further investigation is warranted to explore the potential mediating roles of anonymity and trust on these differential privacy concerns. For example, privacy concerns might be lower for anonymous data activities than for identified ones—people may have similar or even higher levels of privacy concerns about sharing with researchers as they do about the app owners collecting the data if the sharing is not anonymous. Relatedly, people who trust different sources more might also express fewer privacy concerns, and so differences observed may be due to greater trust in research than the technology companies themselves.

Not surprisingly, similar types of data that participants expressed privacy concerns about were those that they were least willing to share with researchers. This might have implications for policies around broad consent for data sharing, whereby participants might need to be given the choice to opt in or out of specific types of data collection and sharing activities rather than simply consenting to share or not share all data. Specifically, these findings suggest that if individuals are given a choice of sharing all data or none, many might select to not share, resulting in low enrollment and high rates of missing data thus biasing the sample and study results. Alternatively, providing options about what to share might, at the very least, allow a more representative sample on some of the types of data (eg, sociodemographics) and could allow for a better estimate of how biased the results are for the types of data not shared. However, this study did not examine the impact of compensation, and further research is needed to examine how compensation might alter participants’ willingness to engage in data sharing; understanding the impact of compensation on data sharing—particularly types of data that participants are otherwise generally unwilling to share—may inform ethical considerations.

Finally, data for this study were collected prior to the recent concerns about data-related and privacy issues on both social networking sites and sexual networking apps [44,45], and replication of the findings in the wake of ongoing privacy-related events is warranted. Future research can and should attempt to understand the magnitude and longevity of the impact of these events on constructs such as trust, privacy concerns, and willingness to share data. In the wake of such events, many technology companies seek to update their privacy policies but may do so with little information on what types of protections are most important to their users—researchers studying privacy in online and mobile technologies both within and outside of research are well-suited to understand and subsequently advise on exactly these types of issues.

### Study Strengths and Limitations

In this study, we considered the use of technology and limited interaction procedures as strength as it facilitated large-scale data collection of individuals with substantially fewer resources than would be possible in a standard research study. However, it also necessitated conducting a very brief survey with a limited number of measures. We used a targeted advertisement with a random chance for incentives along with rigorously implementing standards for confirming the veracity and uniqueness of participants to reduce the likelihood of false and duplicate participants [41]. However, in online studies such as this, some degree of duplication or invalid response is likely. We reviewed the terms of service and other policies for several existing social media and sexual networking sites and apps while developing our measures to contextualize them appropriately. Nonetheless, the study constraints limited our ability to ask about the extent to which participants have ever read these policies, and the extent to which they realize that many of the data types and collection activities assessed were those that they have generally agreed to for apps and sites they regularly use remains unclear. We adjusted for rather than focusing on the role of sociodemographic and behavioral factors and future research is warranted to explore how trust, privacy, and willingness to share data might differ according to factors such as HIV status and race/ethnicity. Finally, conclusions regarding trust in this study and concerns about privacy are slightly limited as this is, by definition, a sample that agreed to participate in an online research study. However, this is also a sample using the apps and sites from which they were enrolled, which were still trusted less than research, suggesting this finding regarding the relative trust could be reliable more generally even if the actual levels of trust are skewed higher by the nature of the sample.

### Conclusions

This study suggests a relatively favorable view of online and mobile research—this large sample of GBMSM across the United States expressed a moderate level of trust in online research and few data-related privacy concerns. Moreover, the sample was nearly evenly split based on their willingness to have several types of app-based data shared with researchers, suggesting the analysis of such data might be potential avenues for future collaborations between researchers and technology companies. The findings highlighted the role of the privacy paradox, as participants expressed concerns about numerous data-related activities that they have likely permitted upon agreeing to use the apps and websites from which they were enrolled. Thus, researchers and ethical boards should consider these moderate levels of trust, privacy concerns, and willingness to share data when evaluating the risks and benefits of such partnerships. Meanwhile, other perspectives, such as legal and technical insights, should also be considered. When researchers can affect decision making, apps used for research purposes should be designed to decrease the extent to which participants must agree to data collection activities that concern them. For example, allowing participants to opt in or out of different aspects and providing multimedia (ie, “gist”) rather than text-based (ie, “verbatim”) explanations of the terms might reduce the privacy paradox in online and mobile research. For any secondary collection of data from apps, researchers should provide potential participants control over the types of data shared to the greatest extent possible, given the varying levels of concerns across different types of data that apps might have access to. Further research in this area is critical, particularly in the light of ongoing public awareness of and debate about technology and privacy [44,45].

## Appendix

Multimedia Appendix 1 Measures of trust, privacy concerns, and data sharing.

## Abbreviations

GBMSM: gay, bisexual, and other men who have sex with men
GPS: global positioning system
LGBT: lesbian, gay, bisexual, and transgender
RMANOVA: repeated measures analysis of variance
