# Supplementary material for: Privacy, Trust, and Data Sharing in Web-Based and Mobile Research: Participant Perspectives in a Large Nationwide Sample of Men Who Have Sex With Men in the United States
Source: J Med Internet Res. 2018 Jul 4;20(7):e233. doi: 10.2196/jmir.9019 (PMC6053617; doi:10.2196/jmir.9019)
Supplement: Multimedia Appendix 1 [file jmir_v20i7e233_app1.pdf]

ONLINE SUPPLEMENTARY MATERIALS
Multimedia Appendix 1: Measures of Trust, Privacy Concerns, and Data Sharing

Trust in Online Research and Services

We are interested in knowing more about how much you trust various organizations and businesses to protect the privacy and confidentiality of the data they collect on you. Please assume you are being asked to provide similar information to each. **How much do you trust that each of the following sources would guard the privacy and confidentiality of your personal information?**

	Not at all trusting	A little trusting	Trusting	Very trusting
An online research study conducted by researchers at a university	1	2	3	4
An online research study conducted by an LGBT community center	1	2	3	4
An online research study conducted by a government health agency such as the Centers for Disease Control (CDC) or your state's Department of Health	1	2	3	4
A mobile networking app for gay/bi men (e.g., Grindr, Scruff)	1	2	3	4
A mobile networking app for the general public (e.g., OKCupid, Tinder)	1	2	3	4
An online shopping website (e.g., Amazon, Overstock)	1	2	3	4
An online email website (e.g., Gmail, Yahoo)	1	2	3	4
An online search engine (e.g., Google, Bing)	1	2	3	4
A research study conducted by researchers at a university in collaboration with a mobile networking app for gay/bi men	1	2	3	4

App-Based Privacy and Confidentiality Threats

Imagine that a new app has been developed and has grown in popularity to be more widely used than those which are currently most popular among gay and bisexual men for finding, chatting with, and meeting nearby guys. The app has many popular features, including: (1) a public profile with photos and basic information about you; (2) a private photo album to put additional photos that you can share with selected people; (3) the ability to interact with other users via chats and likes; (4) a private Match Survey with questions to help the app prioritize matches for you; (5) a grid displaying other users that allows you to sort by several features, including distance and match quality; (6) health promotion campaigns, such as reminders to get and information on where to receive testing and treatment for HIV and/or other STIs; (7) custom alerts about things you may care about such as when a favorite user or a person with a high match to you is near your location; (8) features to let you track your interactions with your connections, such as how you know each other and if/when you've had sex with them.

The questions below each contains a variety of types of information that will be accessible to the app owners. We're interested in knowing how you feel about the extent to which each type of information could be a threat to your privacy. We will ask you about activities involving these forms of information in three categories: (1) collecting and storing these data [*to improve, tailor, and develop the services you use**]; (2) selling these data anonymously to third-party marketing groups [*so they can develop ads that are relevant and interesting to you**]; and (3) sharing these data anonymously with researchers [*to improve the health of your community**]. Please assume that all of these activities are allowable based on the Terms of Service and Privacy Policy of the app, and as such none of them are optional.
Which of the following concern you as a threat to your privacy? Check the boxes for those that *concern* you and leave them blank for those that don't.

EXPERIMENTAL MANIPULATION: Text presented above [in italics font within brackets] was randomly shown to 50% of participants and hidden from the other 50% as an experimental manipulation.

	**App owners privately collecting and storing**	**App owners selling anonymously to third-party companies**	**App owners sharing anonymously with researchers**
Information you put in your public profile, such as your age, height, and relationship status	[ ]	[ ]	[ ]
Information you provide privately to create your account, such as your birthdate and zip code	[ ]	[ ]	[ ]
Information you provide to the app to help find better matches, such as your HIV status or sexual/dating interests	[ ]	[ ]	[ ]

Information about your mobile device, such as its operating system and service provider	[ ]	[ ]	[ ]
Information about who you interact with in the app, such as anonymous demographic information on who you chat with	[ ]	[ ]	[ ]
Information on how you use the app, such as how often you login	[ ]	[ ]	[ ]
Information on whether you participate in any of the app's health promotion campaigns, such as reminders to get an HIV test	[ ]	[ ]	[ ]
Information gathered from your device's GPS regarding where you've used the app	[ ]	[ ]	[ ]
Information gathered from your device's operating system about what other apps you have on your phone	[ ]	[ ]	[ ]
Information gathered by the app for its marketing campaigns, such as whether you've clicked on different advertisements in the app	[ ]	[ ]	[ ]
Information the app gathers from its advertising partners when you leave the app after clicking on one of their ads, such as how you utilized their services	[ ]	[ ]	[ ]
Information the app gathers or can generate about you when you use the app, such as what ads you're most likely to respond to or the types of people you seem to match best with	[ ]	[ ]	[ ]

Consent for Research Data Sharing

Within this study, we are not gathering any data on you from any apps or sites that you use. However, please imagine we were interested in connecting data collected by the app with the data you provided in

this survey. **Which of the following would you give us permission to gather anonymously from the app owners to link with your survey data?**

	Definitely not	Probably not	Probably	Definitely
Information you put in your public profile, such as your age, height, and relationship status	1	2	3	4
Information you provide privately to create your account, such as your birthdate and zip code	1	2	3	4
Information you provide to the app to help find better matches, such as your HIV status or sexual/dating interests	1	2	3	4
Information about your mobile device, such as its operating system and service provider	1	2	3	4
Information about who you interact with in the app, such as anonymous demographic information on who you chat with	1	2	3	4
Information on how you use the app, such as how often you login	1	2	3	4
Information on whether you participate in any of the app's health promotion campaigns, such as reminders to get an HIV test	1	2	3	4
Information gathered from your device's GPS regarding where you've used the app	1	2	3	4
Information gathered from your device's operating system about what other apps you have on your phone	1	2	3	4
Information gathered by the app for its marketing campaigns, such as whether you've clicked on different advertisements in the app	1	2	3	4
Information the app gathers from its advertising partners when you leave the app after clicking on one of their ads, such as how you utilized their services	1	2	3	4
Information the app gathers or can generate about you when you use the app, such as what	1	2	3	4

ads you’re most likely to respond to or the types of people you seem to match best with				
